# Antioxidant Capacity and Accumulation of Caffeoylquinic Acids in *Arnica montana* L. In Vitro Shoots After Elicitation with Yeast Extract or Salicylic Acid

**DOI:** 10.3390/plants14060967

**Published:** 2025-03-19

**Authors:** Maria Petrova, Maria Geneva, Antoaneta Trendafilova, Kamelia Miladinova-Georgieva, Lyudmila Dimitrova, Mariana Sichanova, Milena Nikolova, Viktoria Ivanova, Margarita Dimitrova, Magdalena Sozoniuk

**Affiliations:** 1 Institute of Plant Physiology and Genetics, Bulgarian Academy of Sciences, Acad. G. Bonchev Street, Bldg. 21, 1113 Sofia, Bulgaria; marry_petrova@yahoo.com (M.P.); boykova2@yahoo.com (M.G.); dim.lyudmila@gmail.com (L.D.); msichanova@abv.bg (M.S.); mstoyadinova@abv.bg (M.D.); 2 Institute of Organic Chemistry with Centre of Phytochemistry, Bulgarian Academy of Sciences, Acad. G. Bonchev Street, Bldg. 9, 1113 Sofia, Bulgaria; antoaneta.trendafilova@orgchm.bas.bg (A.T.); viktoria.genova@orgchm.bas.bg (V.I.); 3 Institute of Biodiversity and Ecosystem Research, Bulgarian Academy of Sciences, Acad. G. Bonchev Street, Bldg. 23, 1113 Sofia, Bulgaria; mtihomirova@gmail.com; 4 Institute of Plant Genetics, Breeding and Biotechnology, University of Life Sciences in Lublin, 20-950 Lublin, Poland; magdalena.sozoniuk@up.lublin.pl

**Keywords:** micropropagation, total phenolic content, total flavonoid content, caffeoylquinic acids, HPLC, antioxidant potential

## Abstract

*Arnica montana* L. is an important herbal medicinal plant that belongs to the family Asteraceae. This plant has been known for its medicinal uses for centuries. *A. montana* exhibits several pharmacological properties, including immunomodulatory, anti-inflammatory, anticancer, antioxidant, and antibacterial effects. For the first time, the impacts of the biotic elicitor yeast extract, and the abiotic elicitor salicylic acid on micropropagation, antioxidant potential, and accumulation of caffeoylquinic acids in arnica in vitro shoots were assessed. The results showed that yeast extract applied at 100 mg/L significantly promotes shoot multiplication, biomass yield, total phenolic content, and synthesis of caffeoylquinic acids compared to control untreated shoots. Flavonoid content was the highest in samples treated with 200 mg/L of yeast extract, although at this concentration the measured biometric parameters began to decrease. Salicylic acid at 100 µM was found to be effective in the induction of vigorous shoots, shoot height growth, and biomass accumulation; nevertheless, this elicitor downregulated the caffeoylquinic acid level, total phenolics, and flavonoids. Increasing the concentration of salicylic acid to 200 µM caused shoot multiplication and fresh biomass accumulation reduction. Both elicitors modulated the activity of antioxidant enzymes against oxidative stress. Overall, the use of these substances can improve the growth and biomass yield in *Arnica* in vitro shoots.

## 1. Introduction

*Arnica montana* L., also known as mountain tobacco, is a medicinal plant that has been used for centuries in European medicine. The plant species belongs to the Asteraceae family and generally grows in nutrient-poor and dry heathlands, shrublands, and grasslands of mountains. *A. montana* is a source of more than 150 biologically active compounds, the majority of which were classified as phenolic compounds (phenolic acids, flavonoids, coumarins, and lignans), terpenoids (monoterpenes, essential oils, sesquiterpene lactones, diterpenes, triterpenes, and carotenoids), pyrrolizidine alkaloids, polyacetylenes, and oligosaccharides [1,2]. Due to habitat loss and extensive harvesting for commercial and medicinal purposes, *A. montana* is listed as a threatened species in the European Red List of Vascular Plants (Least Concern) [3] and in the Red Data Books and Red Data Lists of many European countries [4].

The use of plant biotechnological methods, especially plant cell and tissue cultures, is crucial for rare and threatened medicinal plant conservation, as it provides an opportunity to overcome the inherent problems of wild herbal products: content variability, toxic components, and impurities [5]. Plant in vitro cultures represent an attractive, cost-effective, and eco-friendly approach for large-scale plant production, reproduction, and protection of species that are difficult to regenerate by conventional methods, and the synthesis of high-value metabolites of commercial interest [6]. Plant biotechnological techniques have many benefits over the cultivation of whole plants in the field: growth cycles are of weeks rather than years; climatic or environmental problems are avoided; manipulation of one or more culture parameters is possible; and elucidation of the intricate mechanisms of secondary metabolite synthesis is facilitated [7,8]. Elicitation is one of the most effective biotechnological tools for modulating and enhancing valuable plant secondary metabolite synthesis. Elicitors are usually compounds of a chemical or biochemical nature that can induce physiological changes in the target living organism and trigger the generation of secondary metabolites by activating defense-related genes involved in their biosynthesis [9]. Salicylic acid (SA) is a phenolic compound present in plants and is recognized as an important signal in plant defense response to biotic and abiotic stress conditions [10]. This plant hormone influences plant growth and development, participates in some signal transduction pathways to induce particular enzymes, and improves secondary metabolites in in vitro plant cultures of various medicinal plants [11]. Exogenous SA treatment upregulated the expression of phenylalanine ammonia-lyase (PAL), an important enzyme in the phenylpropanoid pathway, resulting in the accumulation of phenolic compounds in *Salvia officinalis* L., *Salvia virgata* Jacq, and *Hypericum perforatum* L. in vitro cultures [12,13]. The biotic elicitor yeast extract (YE), rich in B-complex vitamins and other crucial compounds such as chitin, N-acetyl-glucosamine oligomers, β-glucan, glycopeptides, and ergosterol, is reported to promote plant growth, initiate plant defense responses, and enhance metabolite synthesis [14]. Several studies have documented a high accumulation of secondary metabolites and the activation of the PAL enzyme by yeast extract, in addition to the in vitro cultured cell suspension or plant tissue culture [15,16].

Plants have a complex antioxidant defense system that includes enzymatic and non-enzymatic antioxidants to combat oxidative damage caused by reactive oxygen species (ROS). Superoxide dismutase (SOD), catalase (CAT), ascorbate peroxidase (APX), and guaiacol peroxidase (GPX) are the main enzymatic antioxidants, while non-enzymatic antioxidants include ascorbate, α-tocopherol, phenolic compounds, flavonoid compounds, and others [17]. One of the most significant secondary metabolite groups in *A. montana* plants is phenolic compounds [1]. Both biotic and abiotic factors have a considerable impact on the quantity and quality of these chemicals. Given the high pharmaceutical importance and widespread use of mountain arnica today, it is crucial to investigate antioxidant defense mechanisms in this plant. In the available literature, there are no reports on the influence of elicitors on secondary metabolism and antioxidant defense systems in arnica.

In order to promote the accumulation of phenolic compounds, this study aimed to evaluate the effects of SA and YE on plant development, caffeoylquinic acid synthesis, and the antioxidative defense system of *A. montana* in vitro shoots.

## 2. Results

### 2.1. Effect of YE on Shoot Organogenesis, Shoot Growth, and Development

**Figure 1:**
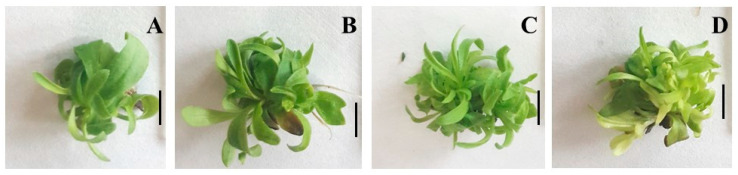
Shoot culture of *Arnica montana* L. after 5 weeks cultivation on MS medium containing 0.5 mg/L BAP and different concentrations of yeast extract (**A**) control without YE, (**B**) 50 mg/L YE, (**C**) 100 mg/L YE and (**D**) 200 mg/L YE. The scale bar represents: 1 cm.

The organogenesis efficiency was 100% on all tested nutrient media. Yeast extract included in the nutrient media positively affected the morphogenetic potential of *A. montana* (Table 1, Figure 1). An increase in the average number of axillary shoots per explant was observed in all tested yeast extract concentrations (50, 100, 200 mg/L) compared to the control plants grown on Murashige and Skoog’s (MS) nutrient medium supplemented only with 6-benzylaminopurine (BAP).

The best results were obtained in plants treated with 100 mg/L yeast extract, where the average number of shoots per explant was the highest (5.2) and the greatest biomass yield (0.58 g FW) was produced (Table 1, Figure 1C). Compared to the *A. montana* growth at the control medium, the shoot height increased when grown on the medium supplied with 50 and 100 mg/L YE. The nutrient medium supplemented with the highest YE concentration (200 mg/L) produced significantly shorter shoots of *A. montana* than the control medium. Some plants grown on medium supplemented with 200 mg/L YE showed signs of chlorosis (Figure 1D). The lowest shoot number per explant (3.20) and the lowest biomass yield were observed on the elicitor-free medium (0.32 g FW). The study demonstrates the benefits of using yeast extract to accelerate the growth potential of arnica plants in vitro.

### 2.2. Effect of YE on Antioxidant Enzyme Activity

**Figure 2:**
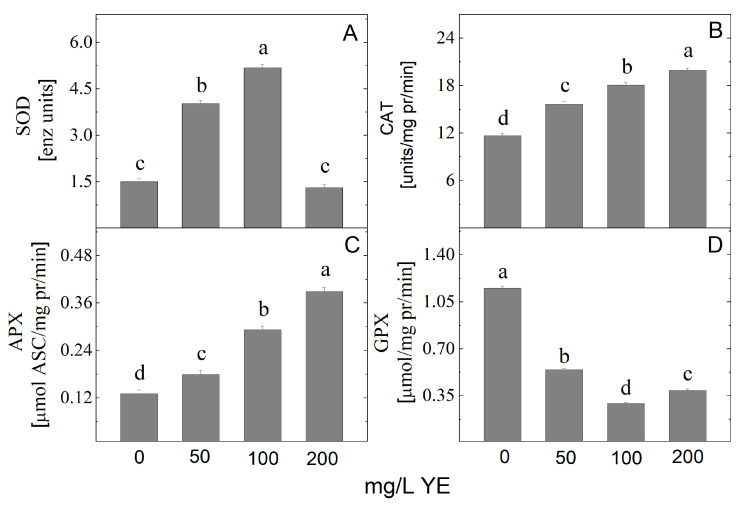
The activity of antioxidant enzymes superoxide dismutase (SOD) (**A**), catalase (CAT) (**B**), ascorbate peroxidase (APX) (**C**), and guaiacol peroxidase (GPX) (**D**) in *Arnica montana* shoots elicited with YE applied at different concentrations (0, 50, 100 and 200 mg/L). Values are means ± SE, n = 20; different letters indicate significant differences assessed by the Fisher LSD test (*p* ≤ 0.05) after performing ANOVA one-way analysis. We used the letter “a” for the highest data value and descended to the next for lower data values.

Elicitation usually triggers stress responses in in vitro plant cultures. The level of antioxidant enzyme activity in the plant is often up-regulated due to elicitor treatment. In the current study, YE altered the antioxidant enzymes SOD, APX, CAT, and GPX activity (Figure 2). The analysis revealed that the extracts from plants treated with YE during in vitro propagation possess higher SOD, APX, and CAT activities in comparison with control cultures. Only plants treated with the highest concentration of YE showed SOD activity close to control (Figure 2A). The highest SOD activity was noted at 100 mg/L YE. The activity of antioxidant enzymes APX and CAT show up-regulated linear dependence with an increase in the concentration of YE. The maximum activity of CAT and APX was obtained at 200 mg/L YE (Figure 2B,C). GPX activity decreased after YE treatment of in vitro cultured *A. montana* plantlets (Figure 2D).

**Figure 3:**
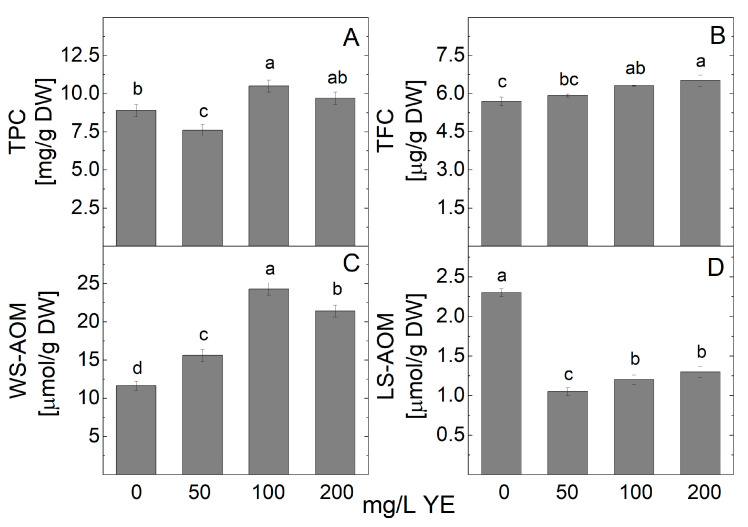
The content of metabolites with antioxidant power (TPC (**A**) and TFC (**B**), WS-AOM (**C**), and LS-AOM (**D**)) in *Arnica montana* shoots elicited with YE applied at different concentrations (0, 50, 100, and 200 mg/L). Values are means ± SE, n = 20; different letters indicate significant differences assessed by the Fisher LSD test (*p* ≤ 0.05) after performing ANOVA one-way analysis. We used the letter “a” for the highest data value and descended to the next for lower data values.

The content of metabolites with antioxidant power in *A. montana* shoot extract was analyzed spectrophotometrically, and the results are presented in Figure 3. An increase in total phenolic content (TPC) in in vitro shoot cultures treated with 100 and 200 mg/L yeast extract was observed (Figure 3A). The highest phenolic content was recorded at 100 mg/L YE (10.5 mg/g DW). The 50 mg/L yeast extract treatment reduced the phenolic content of the shoot culture compared to the control plants. The total flavonoid content (TFC) of *A. montana* plantlets was also affected by YE added to the MS nutrient medium. The highest TFC was achieved with the 200 mg/L YE treatment (6.5 mg/g DW), followed by the 100 mg/L YE treatment, which resulted in 6.3 mg/g DW (Figure 3B).

The data on the spectrophotometric quantification of the water-soluble (WS-AOM) and the lipid-soluble (LS-AOM) metabolites with antioxidant capacity, expressed as equivalents of ascorbate and α-tocopherol, are shown in Figure 3C,D. The content of WS-AOM was found to be promoted by the addition of YE in the MS nutrient media. The highest level of WS-AOM was recorded at 100 mg/L YE (Figure 3C). The LS-AOM decreased at all tested YE concentrations (Figure 3D).

**Figure 4:**
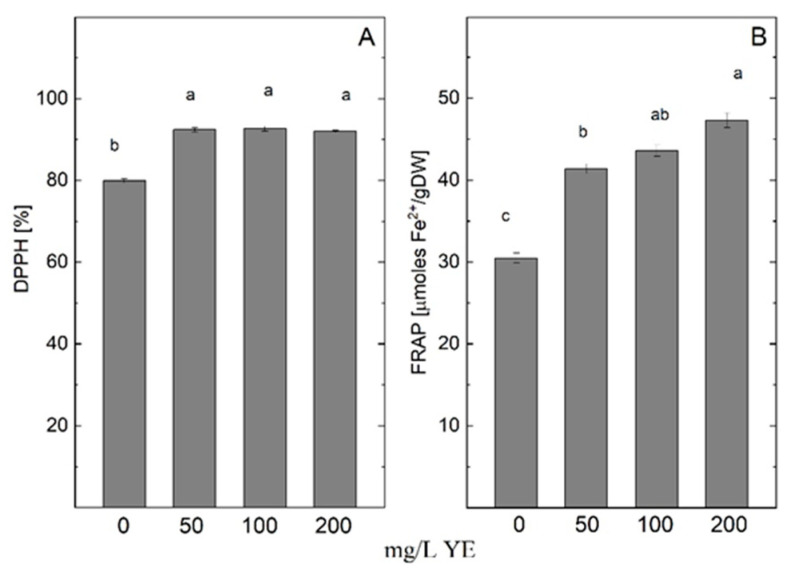
Antioxidant potential—DPPH free radical scavenging activity (**A**) and ferric-reducing antioxidant power (FRAP) (**B**) in *Arnica montana* shoots elicited with YE applied at different concentrations (0, 50, 100, and 200 mg/L). Values are means ± SE, n = 20; different letters indicate significant differences assessed by the Fisher LSD test (*p* ≤ 0.05) after performing ANOVA one-way analysis. We used the letter “a” for the highest data value and descended to the next for lower data values.

Total antioxidant capacities assessed by 2,2-diphenyl-1-picrylhydrazyl (DPPH) free radical scavenging activity (DPPH method) and ferric-reducing antioxidant power (FRAP method) showed an increment level in shoots treated with yeast extract (Figure 4). The methanolic extract from the shoots grown on MS medium containing 200 mg/L YE exhibited the highest activity assessed by FRAP assay (47.297 µmol Fe^2+^/gDW). At the same time, the plantlets treated with all tested YE concentrations showed higher activity recorded by the DPPH method compared with control plants.

### 2.3. Identification of the Main Secondary Metabolites in A. montana Shoots

**Figure 5:**
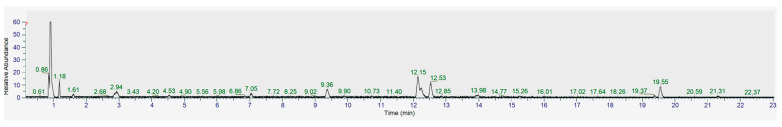
UHPLC-MS base peak chromatogram of *A. montana* shoot extract in negative mode.

The UHPLC-MS/MS analysis in a negative ionization mode was used to identify the main secondary metabolites in *A. montana* shoots (Figure 5, Table 2; Figure S1). Thus, out of 23 compounds, 8 compounds were unambiguously identified by direct comparison with authentic standards, and 15 compounds were tentatively identified based on their retention time, *m*/*z* values, molecular formula, and fragmentation pattern and comparison with the data described in the literature and open-access LC-MS libraries. The pairs **13**/**16** and **20**/**22** displayed [M-H]^−^ at *m/z* 601 and *m/z* 763, respectively. Their MS/MS showed fragment ions at *m/z* 395 and *m/z* 557 [M-H-Caffeoyl-COO]^−^, *m/z* 353 and *m/z* 515 [M-H-Caffeoyl-86]^−^, and *m/z* 233 and *m/z* 395 [M-H-2Caffeoyl-COO]^−^, respectively, and *m/z* 191 (quinic acid), suggesting a structural type of di- and tricaffeoyl quinic acids (DCQA and TCQA), which contain an additional acyl group with molecular formula C_3_H_3_O_3_ (86 Da). Based on the literature review, two possibilities were proposed for this group—methoxyoxaloyl [18,19] or malonyl [20,21]. A careful inspection of the published data revealed that distinguishing between these acyl groups is a difficult task, as they show the same fragment ions in their MS. Furthermore, the comparison of our data with those published for either methoxyoxaloyl or malonyl DCQA and TCQA did not allow for their unambiguous identification.

As can be seen from Table 2, caffeoylquinic acids (CQAs) (**6**, **7**, **9**, **10**, **12**, **13**, **15–20** and **22**) were the main class of metabolites detected in *A. montana* shoots followed by hydroxybenzoic acid glycosides (**2**–**5**) and flavonoids (**11**, **14**, **21** and **23**).

### 2.4. Content of Caffeoylquinic Acids After Elicitation with YE

**Figure 6:**
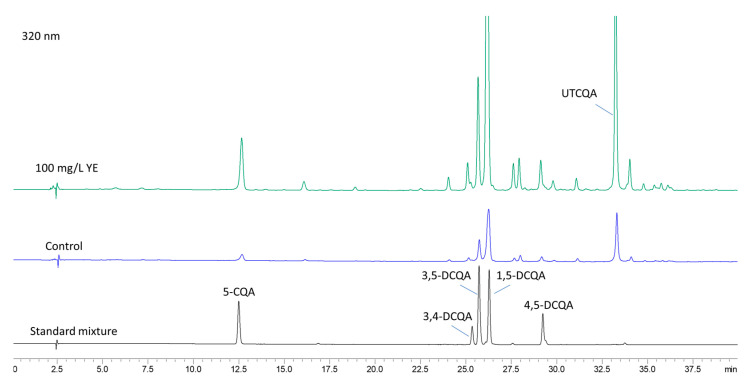
HPLC chromatogram at 320 nm of a standard mixture of CQAs, control, and sample treated with 100 mg/L YE.

Based on the results from UHPLC-MS/MS and HPLC-DAD (Figure 6) analyses, six caffeoylquinic acids were chosen for monitoring the impact of elicitors. The content of chlorogenic (5-CQA), 3,4-, 1,5-, 3,5- and 4,5-DCQA in *A. montana* shoots was determined from the regression equations of the corresponding calibration curves, while the quantity of the undetermined tricaffeoylquinic acid derivative (UTCQA) was calculated as mg equivalents of 1,5-DCQA per g DW.

The results from the quantitative determination of the six caffeoylquinic acids (Table 3) showed that 1,5-DCQA (1.34–3.02 mg/g DW) was the most abundant compound, followed by UTCQA (0.74–1.74 mg/g DW), 5-CQA (0.23–0.68 mg/g DW), 3,5-DCQA (0.35–0.61 mg g^−1^ DW), 4,5-DCQA (0.09–0.21 mg/g DW), and 3,4-DCQA (0.08–0.17 mg/g^−1^ DW). The amount of all identified compounds increased after YE treatment. The highest content of caffeoylquinic acids was detected in shoots treated with 100 mg/L YE, which was over 2-fold greater (total amount of 6.45 mg/g DW) than in control shoots (total amount of 3.02 mg/g DW). The increase in YE concentration to 200 mg/L led to a decline in the content of all compounds.

### 2.5. Effect of SA on Shoot Organogenesis, Shoot Growth, and Development

**Figure 7:**
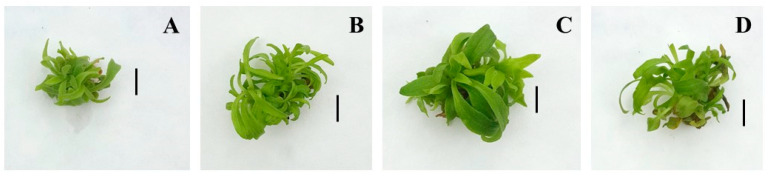
Shoot culture of *Arnica montana* L. after 5 weeks of cultivation on MS medium containing 0.5 mg/L BAP and different concentrations of salicylic acid (**A**) control without SA, (**B**) 50 µM SA, (**C**) 100 µM SA, and (**D**) 200 µM SA. The scale bar represents: 1 cm.

The mean number of shoots produced per explant on media supplemented with 50 µM SA and 100 µM SA did not differ statistically from this record on an SA-free medium (Table 4, Figure 7). A decrease in the number of shoots was observed at the highest concentration of SA (200 µM). The shoots induced on SA-containing media showed better growth in height and greater biomass accumulation compared to those cultured on the control medium without SA. Maximum shoot fresh biomass was achieved after treatment with 100 µM SA (Table 4). The plants derived from this treatment had bigger leaves with large leaf petioles and a larger leaf area (Figure 7C). Control plants were the shortest in height and accumulated the least fresh biomass. Compared to the control plants, the plantlets grown on MS medium containing SA at all studied concentrations were vigorous and dark green for the entire culture period.

### 2.6. Effect of SA on Antioxidant Activity of Arnica montana In Vitro Shoots

**Figure 8:**
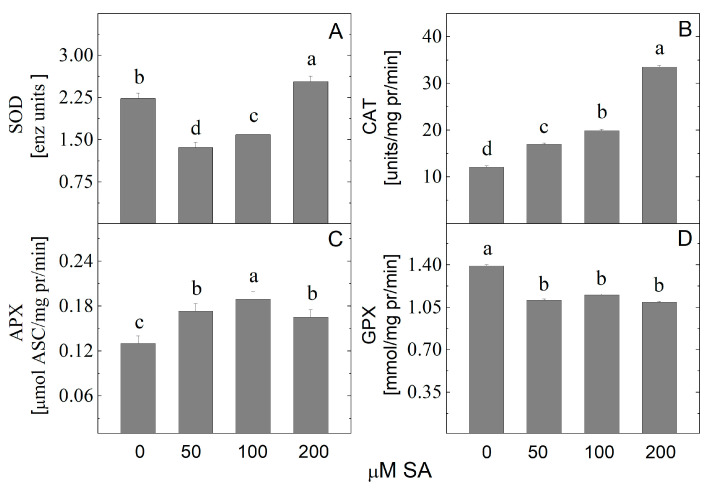
The activity of antioxidant enzymes superoxide dismutase (SOD) (**A**), catalase (CAT) (**B**), ascorbate peroxidase (APX) (**C**), and guaiacol peroxidase (GPX) (**D**) in *Arnica montana* shoots elicited with SA applied at different concentrations (0, 50, 100 and 200 µM). Values are means ± SE, n = 20; different letters indicate significant differences assessed by the Fisher LSD test (*p* ≤ 0.05) after performing ANOVA one-way analysis. We used the letter “a” for the highest data value and descended to the next for lower data values.

Antioxidant enzyme spectrophotometric measurements revealed that *A. montana* plantlets treated with SA had altered levels of all studied enzymes (Figure 8). Plantlets treated with 200 µM SA showed increased SOD activity. The SA gradually raised the CAT activity, with the highest value recorded in the shoots treated with 200 µM SA. The APX activity also increased after SA treatment. The highest APX enzyme activity was measured when plantlets were cultured on a medium containing 100 µM SA. The plantlet’s GPX activity decreased due to the SA application.

**Figure 9:**
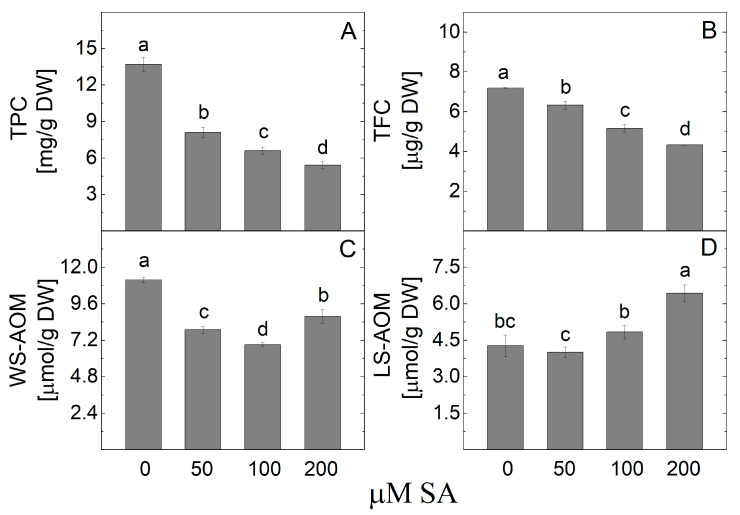
The content of metabolites with antioxidant power (TPC (**A**) and TFC (**B**), WS-AOM (**C**), and LS-AOM (**D**)) in *Arnica montana* shoots elicited with SA applied at different concentrations (0, 50, 100, and 200 µM). Values are means ± SE, n = 20; different letters indicate significant differences assessed by the Fisher LSD test (*p* ≤ 0.05) after performing ANOVA one-way analysis. We used the letter “a” for the highest data value and descended to the next for lower data values.

The analysis of the TPC levels in the arnica shoot extract showed that their content decreased after the salicylic acid treatment (Figure 9). The lowest TPC was detected in plant samples derived from the 200 µM SA treatment. The same trend was observed in the TFC, which also showed a reduction compared to control plants without SA addition. It was found that the WS-AOM level decreased after the application of SA, while the LS-AOM content increased due to SA treatment.

**Figure 10:**
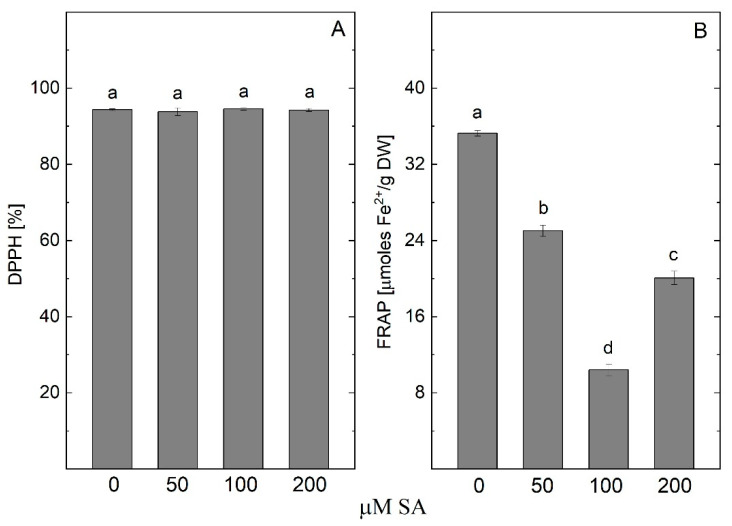
Antioxidant potential—DPPH free radical scavenging activity (**A**) and ferric-reducing antioxidant power (FRAP) (**B**) in *Arnica montana* shoots elicited with SA applied at different concentrations (0, 50, 100 and 200 µM). Values are means ± SE, n = 20; different letters indicate significant differences assessed by the Fisher LSD test (*p* ≤ 0.05) after performing ANOVA one-way analysis. We used the letter “a” for the highest data value and descending to the next for lower data values.

The antioxidant activity, measured by the FRAP method in the *A. montana* plantlets, was significantly decreased by treatment with SA (Figure 10). The DPPH activity did not show a significant difference in the treated plants compared to the control.

### 2.7. Content of Caffeoylquinic Acids After Treatment with SA

**Figure 11:**
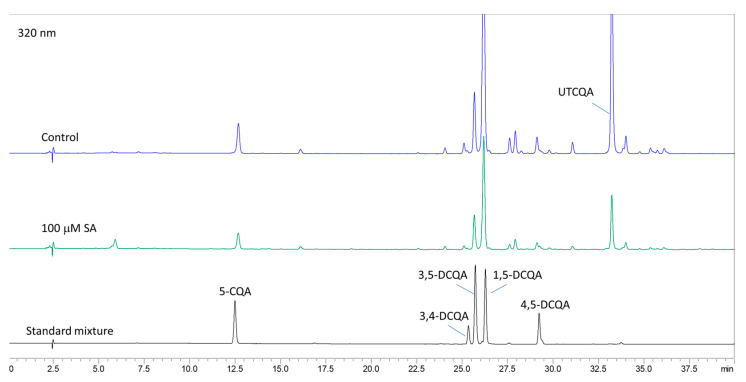
HPLC chromatogram at 320 nm of a standard mixture of CQAs, control, and sample treated with 100 µM SA.

The results of the HPLC quantitative determination of the content of CQAs in shoots treated with SA (Figure 11) are presented in Table 5. The total content of caffeoylquinic acids and the individual detected components decreased with increasing SA concentration. 5-CQA varied from 0.19 to 0.39 mg/g DW in the samples. The content of dicaffeoylquinic acids dominated in samples, similar to the results after YE treatment. The most abundant compound was 1,5-DCQA with values from 0.66 to 2.07 mg/g DW. The lowest 5-CQA, DCQAs, and UTCQA content was found in shoots treated with the highest SA concentration (200 µM).

## 3. Discussion

Biotechnological approaches, including in vitro cell and tissue cultures, represent an alternative to the wild-grown medicinal plant collection or traditional agronomic farming for the large-scale production of secondary metabolites [26,27]. They are a practical and flexible tool for elucidating processes within the plant organism connected to the secondary metabolites production [28]. By using them, it is possible to increase the growth and productivity of plant cells and to automate and control these processes. The fact that in vitro cultures are more “metabolically viable” than in vivo grown plants is also significant. Elicitation is a promising strategy that can be used to enhance secondary metabolites in plant in vitro culture. An elicitor can activate any plant defense mechanism, supporting secondary metabolism to defend the cell and the plant as a whole. When applied in a small amount to a living cell system, it initiates or accelerates the production of certain substances [29]. Numerous factors, such as elicitor concentration, selectivity, duration of exposure, age of culture, cell line, growth regulation, nutrient composition, and cell wall material quality, affect the secondary metabolite synthesis [30]. To the best of our knowledge, studies on the effects of elicitors YE and SA on the levels of secondary metabolite content in micropropagated *A. montana* have not been conducted to date. However, available literature studies confirm the effectiveness of these substances in increasing the secondary metabolite biosynthesis in plant cell tissue and organ cultures of other members of the Asteraceae family [31].

### 3.1. Effect of Yeast Extract on Growth and Antioxidant Defense System

The yeast extract is frequently applied because it is a relatively inexpensive and accessible biotic elicitor, making it a practical choice for large-scale plant cell and tissue culture systems. The yeast extract is used as a supplement to boost plant growth due to its high content of amino acids, nutrients, vitamins, and minerals [32,33]. However, the presence and concentration of yeast extract in the nutrient medium possess various effects on different plant species. It has been established that adding a lower yeast extract concentration to the medium is beneficial, whereas applying a higher concentration inhibits growth [34]. An enhanced multiplication and *A. montana* plantlets’ growth rate in terms of shoot height, number of shoots per explant, and biomass after treatment with yeast extract were achieved in the current study. The highest biomass accumulation and shoot production were obtained after 100 mg/L yeast extract application. An increase in shoot multiplication and biomass accumulation was observed in shoots elicited with YE in other medicinal plants such as *Plumbago indica* L. [35], *Stevia rebaudiana* Bertoni [34], and *Pueraria tuberosa* (Roxb. Ex Willd.) DC [36]. Some authors reported the negative effect on biomass production in *Curcuma mangga* Val., *Knautia sarajevensis* (Beck) Szabó, and *Thymus lotocephalus* G. Lopez and R. Morales shoot cultures, as well as morphological abnormalities expressed as growth retardation, chlorosis, and leaf development inhibition after YE application [37,38,39,40]. Thus, it showed that the YE effect on shoot multiplication and biomass production is species-specific, variable, and concentration-dependent. To prevent oxidative stress and cell death by inducing the hypersensitive response caused by excessive elicitor levels, it is necessary to determine the optimal concentration of elicitors [41].

The application of elicitors induces oxidative stress in in vitro plant culture. Thus, they trigger antioxidant enzymes like SOD, CAT, APX, and GPX to detoxify the ROS [42]. In the current study, increased activities of the enzymes with antioxidant potential (SOD, CAT, APX) as a result of the YE treatment were recorded. Gholami et al. [43] also reported an enhanced activity of SOD, CAT, APX, and GPX in field-grown cowpea (*Vigna unguiculata* L.) sprayed with YE (12 g/L). Increased CAT and GPX activities were shown in tomato (*Solanum lycopersicum* L.) after foliar application of YE in concentrations of 3.0, 6.0, and 9.0 g/L, and the effect was dose-dependent [44]. According to Abbas [45], the positive impact of yeast extract may be due to its influence on photosynthetic pigments, phytohormones, and the activity of other enzymes.

The observed higher SOD, CAT, and APX activities of *A. montana* plantlets correlates with the increase in total antioxidant activity measured by FRAP and DPPH methods, as well as with the larger amounts of non-enzymatic antioxidants, in particular TPC, TFC, and WS-AOM. Treatment of purple basil callus culture, as well as cell suspension of apples and flax with YE, has also resulted in significantly enhanced antioxidant power measured by FRAP and DPPH radical scavenging activity, and this was accompanied by a higher accumulation of phenolic compounds and flavonoids [46,47,48].

Phenolics and flavonoids are secondary plant constituents that possess multispectral biological effects, including antioxidant, free radical scavenging abilities, inhibition of hydrolytic and oxidative enzymes, anti-inflammatory, anticarcinogenic, antibacterial, hypolipidemic, antimutagenic, and other activities [36,49,50]. Several publications have reported the antioxidant activity and total polyphenol content of *A. montana* extracts from callus and shoot cultures [51,52,53]. No studies were found about the effect of elicitors on the antioxidant activity of in vitro cultivated *A. montana*. In order to enhance phenolic production, YE at increasing concentrations (50–200 mg/L) was applied in the shoot culture of *A. montana*. The treatment with 100 mg/L YE increased the levels of TPC (10.5 mg/g DW). Using the same amount of YE (100 mg/L) resulted in an enhanced accumulation of phenols in both the hairy root culture of *Aster scaber* L. and the callus culture of basil [48,54]. Enhanced TPC and TFC were obtained by elicitation with YE in *Salvia virgata* Jacq [55], *Thymus lotocephalus* G. Lopez and R. Morales [38], and *Knautia sarajevensis* (Beck) Szabó [39]. The other authors observed no significant differences in TPC between the elicited and control *Origanum majorana* L. plants [56]. Higher secondary metabolite production and PAL activity induction in in vitro-grown *Glehnia littoralis* shoots and roots due to YE treatment have also been reported [40]. It has been proposed that the concentrations of cations such as Ca^2+^, Co^2+^, and Zn^2+^ in YE may be responsible for the elicitor’s stimulating action on secondary metabolite production [57].

### 3.2. Effect of YE on Caffeoylquinic Acids Content

Specialized bioactive metabolites called caffeoylquinic acids (CQAs), which are esters of caffeic acid and quinic acid, are produced via the phenylpropanoid biosynthesis pathway in plants and are found abundantly in the members of the Asteraceae family [58]. CQAs play a protective role against biotic or abiotic stress [59]. They possess a variety of biological activities, including antioxidant, anti-inflammatory, anti-HIV, and antihepatotoxic characteristics, inhibit mutagenesis and carcinogenesis, and slow the aging process [60,61,62,63].

Various CQAs have been detected in *A. montana* extracts by HPLC/MS analysis [18,19,58]. Garcia-Oliveira et al. [64] characterized phenolic compounds in the ethanol extract of *A. montana* dried flowers through HPLC-MS/MS and established that TPC was 27.34 mg/g, with the most prevalent compounds being the dicaffeoylquinic acid isomers (accounting for 79.5% of the TPC). The measured total amount of CQAs in examined shoot samples was in the range (3.02–6.45 mg g), with about 60% of the dicaffeoylquinic acid predominating. Regarding the content of the individual compounds, 1,5-DCQA was found to be the major component in the aerial parts of *A. montana* subsp. *montana* (11.80 mg/g DM), along with 3,5-DCQA (7.52 mg/g DM) and 5-CQA (3.63 mg/g DM) [58]. In another study, 1,5-DCQA was also the major component in the flowers of *A. montana* from Poland, and its amount was found to be 624 mg/g DW, followed by 5-CQA (328 mg/g DM), 3,5-DCQA (165 mg/g DM), and 4,5-DCQA (22.1 mg/g DW) [65]. Some authors reported 3,5-DCQA as the major phenolic acid derivative in *A. montana* L. fresh plants from Poland (450 mg/100 g DW) [66], *A. montana* cultivar ARBO cultivated at different altitudes in Austria (0.54–1.10%) [1], *A. montana* flowers from North Italy (4.9–11.3 mg/g DW) [67], and from Spain (2.87–10.79 mg/g) [68]. The other abundant compounds in these studies were 5-CQA and 1,5-DCQA, while 4,5-DCQA was usually detected in very low amounts. In addition, 1-methoxyoxaloyl-3,5-DCQA has been found in significant amounts (0.24–0.60%) [1].

In this study, six CQAs were detected in the methanol extracts of *A. montana* shoots, with 1,5-DCQA as the major compound. To the best of our knowledge, this is the first report on the qualitative and quantitative determination of CQAs in *A. montana* shoots and on the content of 3,4-DCQA and UTCQA determination. Further, our results showed that YE stimulated the production of CQAs and led to an increase in their levels up to a concertation of 100 mg/L, followed by a decrease at a concertation of 200 mg/L. Similar results have been recently observed for liquid adventitious root cultures of *Inula crithmoides* L. [69]. The positive effect of YE on the accumulation of chlorogenic acid in various plant in vitro cultures (*Malus* × *domestica* Borkh and *Malus pumila* cv. Annurca cell cultures) has already been described [70,71].

### 3.3. Effect of Salicylic Acid on Growth and Antioxidant Defense System

Salicylic acid is an endogenous regulatory signal molecule that promotes the synthesis of secondary metabolites and confers disease resistance in plants [72]. The impact of the exogenous application of SA on plant growth depends on its concentration and the plant species [73]. According to Kovácik et al. [74], for instance, 50 μM SA considerably increased the growth of chamomilla rosette leaves and roots by 32% and 65%, respectively, while 250 μM SA greatly reduced it by 40% and 43%, respectively. In our study, SA showed a beneficial effect on the growth of *A. montana* shoots under in vitro conditions expressed with increasing biomass accumulation and plant height. Applying 100 µM SA to the medium resulted in vigorous shoots and larger, deeper-green leaves, which may have been caused by a higher chlorophyll content. Contrary to our results, the other authors observed that SA treatment caused the development of extremely thin and short shoots with short internodes and small leaves of micropropagated *Stevia rebaudiana* [75].

One of the main pathways by which SA exerts its action is redox signaling and antioxidant defense system regulation [76]. In our study, the activity of CAT and APX increased after SA treatment. SOD activity was upregulated at the highest concentration of SA. SOD, GPX, and CAT activity also significantly increased after the treatment of *Salvia miltiorrhiza* cell culture with SA, and different times and appropriate SA concentrations were needed to reach maximum activity [77]. SA induced the synthesis of important antioxidant enzymes such as SOD, APX, and glutathione reductase, as well as non-enzymatic antioxidants such as ascorbic acid (AsA) and glutathione, all of which reduce ROS accumulation and lipid peroxidation in arsenic-stressed plants [78]. Total phenolic content, DPPH free radical scavenging capacity, SOD, and GPX activities, particularly those of SOD2 and GPX1 isoforms, have increased, while CAT activity has been reduced by the SA addition in a woody plant nutrient medium in micropropagated *Stevia rebaudiana* [79]. Exogenous application of 1 mM SA led to increased H_2_O_2_ content and activities of SOD, APX, polyphenol oxidase, and GPX in *Carthamus tinctorius* L., while the CAT activity did not change [80]. It was revealed that SA mitigates stress by modulating ROS homeostasis [81,82].

Depending on its concentration and specific environmental conditions, SA plays opposing roles as a pro-oxidant (ROS accumulation) and an antioxidant (ROS scavenging). At low concentrations, it stimulates ROS production and, hence, the ROS-mediated defense response; while at high levels, it induces ROS overaccumulation and oxidative stress [76].

SA negatively affected DPPH radical scavenging activity in *Silybum marianum* callus culture [83]. The present study also monitored the total antioxidant potential (DPPH and FRAP assay) reduction in the *A. montana* shoot extracts after the SA treatment.

Some studies have found that SA treatment increases the TPC and TFC levels in medicinal plants [11,77]. Dong et al. [77] reported a higher accumulation of phenolic acids (salvianolic acid B and caffeic acid) in *Salvia miltiorrhiza* cell culture after SA treatment. In our research, the non-enzymatic antioxidant activity of *A. montana* in vitro shoots measured by the TPC and TFC significantly decreased by adding the elicitor salicylic acid to the MS medium. Similar results were observed from Gadzovska et al. [12], who studied the effect of SA elicitation on the accumulation of phenylpropanoids in *Hypericum perforatum* L. shoots, callus, and cell suspension cultures. The phenolic levels in SA-elicited shoots have been constant or even lower than those in the equivalent controls, while in callus and suspension cultures, the phenolic levels increased. The authors conclude that the level of differentiation has participated in the SA response in terms of enrichment in phenolic compounds. The day of a harvest of spinach leaf samples after SA treatment is relevant to the reported TPC and TFC [84]. It has been established that the enhancement continued till the 10th day, after which a slight reducing tendency was noted at high concentrations. In the current study, the samples were collected after 35 days of cultivation in an MS medium containing different concentrations of SA, which may be a reason for the lower amount of TPC detected. Salicylic acid increases the level of nitrogen in the plants. Nitrogen (N) is a compound of many organic substances, including proteins, amino acids, coenzymes, nucleic acids, and chlorophyll [85]. Excessive nitrogen fertilization led to a significant decrease in antioxidant activity and TPC and TFC [86]. The peppermint (*Mentha piperita* L.) leaf’s total phenolic and soluble carbohydrate content was reduced as a result of a high rate of nitrogen and a high concentration of SA applied [87]. Gabr et al. [83] reported lower TPC in the *Silybum marianum* L. callus culture elicited with SA than that obtained from the control. SA has been reported to inhibit ethylene production depending on the SA concentrations used and the plant material analyzed. However, scientists still do not fully understand the ethylene regulation complex network that underlies secondary metabolite accumulation and production [88].

### 3.4. Effect of SA on Caffeoylquinic Acids Content

In this study, the elicitor SA did not stimulate the biosynthesis of CQAs and their content decreased compared to the control. Similarly, Ncube et al. [89] reported that 3,5-DCQA and other CQAs were downregulated as a consequence of SA treatment, while the levels of dicaffeoylquinic acids in globe artichoke (*Cynara cardunculus*) were not significantly affected by the increased concentration of SA [90].

## 4. Materials and Methods

### 4.1. Plant Material

*Arnica montana* seeds were collected from the experimental plots of the Department of Industrial and Medicinal Plants of the University of Life Sciences in Lublin (Poland). Seeds were disinfected using a combination of 70% ethanol and 50% bleach solution (commercial bleach containing 4.85% sodium hypochlorite) and washed three times in sterilized distilled water. The aseptic seeds were germinated on an MS nutrient medium [91] containing 3% (*w*/*v*) sucrose and 0.6% (*w*/*v*) agar. In order to obtain multiple shoots, the stem segments from seedlings were grown on an MS medium supplemented with 1 mg/L BAP and 0.1 mg/L indole-3-acetic acid. The in vitro cultures were maintained according to conditions described by Petrova et al. [92]. The medium pH was adjusted to 5.8 before autoclaving at 121 °C for 20 min.

### 4.2. Elicitor Preparation and Culture Conditions

Control shoots were grown on an MS nutrient medium supplemented with 0.5 mg/L BAP, 3% (*w*/*v*) sucrose, and solidified with 0.6% (*w*/*v*) agar without elicitor. Yeast extract (50, 100, or 200 mg/L) was added to the medium mentioned above before autoclaving. The stock solution of SA was filter-sterilized through a 0.22 µm syringe Millipore filter (Minisart^®^ Sartorius, Goettingen, Germany), and then added to the autoclaved MS medium containing 0.5 mg/L BAP aseptically at the concentrations (50, 100 or 200 µM). MS nutrient medium, BAP, YE and SA were purchased from Duchefa Biochemie B.V, Haarlem, The Netherlands. Explants (nodal segments 1.0 cm long) were cultured in sterilized glass tubes containing 6 mL of nutrient medium. Twenty explants were placed on each medium variant, and each treatment was repeated twice. After 5 weeks the best treatment was selected based on the mean number of shoots per explant, mean height, and mean fresh weight.

All cultures were maintained in a controlled growth chamber at 22 ± 2 °C and 16:8 h light/dark photoperiod under illumination (40 μmol m^−2^ s^−1^) provided by Philips 36 W cool white fluorescent tubes.

### 4.3. Antioxidant Capacity Assays

All solvents were of analytical grade. Ascorbate, guaiacol, hydrogen peroxide, DPPH, and ammonium molybdate were obtained from Merck (Darmstadt, Germany). Nitroblue tetrazolium, riboflavin, and methionine were purchased from Sigma (Jefferson, MO, USA). All other chemicals were of analytical grade.

For protein and antioxidant enzymes, 0.5 g FW frozen plantlet samples were homogenized in 5 mL of 0.1 M phosphate buffer (pH 7.8) containing 2 mM EDTA, 2% (*w*/*v*) polyvinylpyrrolidone, 10% glycerol, and 1 mM phenyl methyl sulfonyl fluoride in an ice bath [93]. The extract was centrifuged at 4 °C for 30 min at 12 500 rpm, and the supernatant was used for the enzyme activities and protein determination.

Total SOD (EC 1.15.1.1) activity assay was based on the spectrophotometrical (560 nm) measurement of inhibition in the p-nitro blue tetrazolium chloride (NBT) photochemical reduction by the enzyme. For the reaction, 3.3 μM riboflavin was added to the reaction mixture containing 50 mM K-phosphate buffer (pH 7.8), 10 mM methionine, 33 μM NBT, 0.66 mM EDTA, and the required amount of enzyme extract. The tubes were placed under fluorescent lamps for 15 min. One unit of SOD activity was defined as the amount of enzyme that causes 50% inhibition of the reduction of NBT [94].

Total CAT (EC 1.11.1.6) activity was measured according to Beers and Sizer [95] by determining the decomposition of H_2_O_2_ (ε = 39.4 mM^−1^ cm^−1^) for 1 min at a reaction mixture containing 100 mM phosphate buffer (pH 7.0), 0.1 mM H_2_O_2_, and 0.1 mL of enzyme extract.

Total APX (EC 1.11.1.1) activity was assayed according to Nakano and Asada [96] by measuring the decrease in the absorbance at 290 nm for 1 min at a reaction mixture consisting of 0.05 mM AsA, 0.5 mM H_2_O_2_, 0.1 mM EDTA, 100 mM phosphate buffer (pH 7.0), and 0.1 mL of enzyme extract.

Total guaiacol peroxidase (GPX, EC 1.11.1.7) activity was determined by measuring the oxidation of guaiacol to tetra-guaiacol per minute, with the increase in absorbance recorded at 470 nm from a 3 mL reaction mixture containing 100 mM phosphate buffer (pH 7.0), 20 mM guaiacol, 0.1 mL of enzyme extract, and 12.3 mM H_2_O_2_ [97].

Soluble protein content was determined according to Bradford [98] by using bovine serum albumin as a standard.

Plant extract obtained from the extraction of air-dried and powdered plant material (0.5–1.0 g) with 80% (*v*/*v*) aqueous methanol was used for TPC, TFC, and the antioxidant capacity determination. The amount of TPC was determined spectrophotometrically using the Folin–Ciocalteu reagent. A standard curve with caffeic acid was used [99].

TFC in plant tissues was determined spectrophotometrically according to Zhishen et al. [100] using the standard curve of catechin. The method is based on the flavonoid–aluminum complex formation, with maximum absorbance at 510 nm. The free DPPH radical scavenging activity was determined spectrophotometrically using the following [101] equation (I%): I% = [(Ablank − Asample)/Ablank] × 100 where I% is the percent inhibition of the DPPH^•^ radical, Ablank and A_sample_—the absorbance of the control reaction (containing all reagents except the extract) and the extract, respectively. The FRAP assay, based on reducing ferric ions (Fe^3+^) to ferrous ions (Fe^2+^), was performed using the method of Benzie and Strain [102].

The levels of WS-AOM and LS-AOM expressed as equivalents of ascorbate and α-tocopherol, respectively, were assessed spectrophotometrically by the method based on the reduction of Mo (VI) to Mo(V) and subsequent recording of the absorption of the formed phosphomolibdenum complex at 695 nm [103].

### 4.4. Preparation of the Samples for Qualitative and Quantitative Analysis of Caffeoylquinic Acids

Briefly, 100 mg of dry plant material was extracted with 2 mL of methanol at room temperature in an ultrasonic bath for 30 min. The extracts were centrifuged, filtered, and transferred in a volumetric flask and made up to 2 mL with methanol. Then, one milliliter of the extract was passed through a solid-phase extraction cartridge, Chromabond^®^ (C18ec, 500 mg, 3 mL, Marchery-Nagel, GMBH&Co., KG, Duren, Germany), to remove chlorophylls. Before analysis, samples were filtered through a 0.22 µm syringe filter.

### 4.5. UHPLC-MS/MS Analysis of the Methanol Extract of A. montana Shoots

UHPLC-MS/MS analysis was performed on a high-resolution Q Exactive Plus^®^ hybrid quadrupole-Orbitrap^®^ mass spectrometer equipped with a heated electrospray ionization source, coupled with a Vanquish UHPLC system (Thermo Fisher Scientific, Bremen, Germany). Chromatographic separation was carried out on an Accucore^TM^ C18 analytical column (150 × 2.1 mm, 2.6 µm) and using 0.1% (*v*/*v*) HCOOH in water (A) and 0.1% (*v*/*v*) HCOOH in CH_3_CN (B) in a gradient mode as described in reference [104].

### 4.6. HPLC-DAD Analysis of Caffeoylquinic Acids

The HPLC analysis was performed on a Schimadzu Nexera-I-LC-2040C 3D Plus liquid chromatograph equipped with a photodiode array detector (Schimadzu, Tokyo, Japan) on analytical column, Force C18 (150 × 4.6 mm, 3 µm), at a temperature of 30 °C. The elution was performed in a gradient mode using a mixture of 0.1% (*v*/*v*) HCOOH in water (A) and methanol (B) as described previously in reference [22]. The injection volume was 4 µL, the flow rate was 0.6 mL/min, and the runs were monitored at 320 nm. Chlorogenic acid (5-CQA), 3,4-DCQA, 1,5-DCQA, 3,5-DCQA, and 4,5-DCQA purchased from Phytolab GmbH & Co. KG (Vestenbergsgreuth, Germany) were used as standards for the calibration curves. The quantity of UTCQA was assessed from the peak area at Rt 33.2 min and calculated as equivalents of 1,5-DCQA. The experiments were performed in triplicate and the results were expressed as mg/g DW.

### 4.7. Statistical Analysis

Data were subjected to a one-way ANOVA analysis of variance for comparison of means, and significant differences were calculated according to the Fisher LSD test at the 5% level using a statistical software package (Statigraphics Plus, version 5.1 for Windows). Data were reported as means ± standard error. Determinations of enzyme activities and metabolite content were performed in three independent experiments.

## 5. Conclusions

The present work reported an elicitor-enhanced metabolite production in *Arnica montana* for the first time. Shoot culture seems to be a promising model system for in vitro accumulation of phenolic compounds, and elicitation is a proven strategy for improving their production yields. Taking into consideration the maximum increase in shoot production/fresh biomass and caffeoylquinic acids accumulation of shoot cultures of *A. montana*, yeast extract (100 mg/L) was the established optimal elicitor. In the current study, salicylic acid is not effective for phenolic compound production. However, more studies are needed in order to find the best SA concentration and exposure time. YE and SA treatments showed an increase in the activities of antioxidant enzymes SOD, CAT, and APX. GPX was downregulated as a result of YE and SA treatment. The findings of this study may serve as a basis for future research into the increase in phenolic compounds with strong antioxidant activity in this highly valued medicinal plant.

## Figures and Tables

**Table 1:** Effect of yeast extract on the growth of *Arnica montana* L. in vitro shoots.

Nutrient Medium	Mean Number of Shoots Explant^−1^	Mean Height cm Shoots^−1^	Fresh Weight g Shoots^−1^
Control	3.20 ± 0.20 ^c^	1.73 ± 0.13 ^ab^	0.32 ± 0.03 ^c^
50 mg/L YE	4.40 ± 0.41 ^ab^	2.09 ± 0.16 ^a^	0.44 ± 0.04 ^b^
100 mg/L YE	5.20 ± 0.45 ^a^	2.10 ± 0.15 ^a^	0.58 ± 0.05 ^a^
200 mg/L YE	4.10 ± 0.38 ^bc^	1.65 ± 0.15 ^b^	0.38 ± 0.03 ^bc^
LSD	1.05	0.42	0.11

The data are presented as means of 20 shoots per treatment variant ± standard error (SE). Different letters indicate significant differences assessed by the Fisher LSD test (*p* ≤ 0.05) after performing one-way ANOVA. We used the letter “a” for the highest data value and descended to the next for lower data values.

**Table 2:** Identification of the compounds in the methanol extract of *A. montana* shoots by UHPC-MS/MS.

No	Rt, min	Compound	Molecular Formula	[M-H]^−^, *m/z*	Δ, ppm	MS/MS Fragments	Ref.
1	0.93	Quinic acid	C_7_H_11_O_6_	191.0555	−3.22	**191** *, 127, 85	[22]
2	1.00	Dihydroxybenzoic acid *O*-hexoside	C_13_H_15_O_9_	315.0732	3.26	315, 153, 152, 109, **108**	[23]
3	1.39	Hydroxy-methoxybenzoic acid *O*-hexoside	C_14_H_17_O_9_	329.0883	1.65	**167,** 152, 123, 108	[24]
4	1.43	Dihydroxybenzoic acid *O*- hexoside	C_13_H_15_O_9_	315.0728	2.09	315, 153, 152, 109, **108**	[23]
5	1.61	Syringic acid *O*-hexoside	C_15_H_19_O_10_	359.0992	2.26	359, **197**, 167, 153, 123	[23]
6	1.83	Neochlorogenic acid (3-*O*-caffeoylquinic acid)	C_16_H_17_O_9_	353.0880	0.89	353, **191**, 179, 135	St
7	2.94	Chlorogenic acid (5-*O*-caffeoylquinic acid)	C_16_H_17_O_9_	353.0884	1.3	353, **191**, 179, 135	St
8	3.86	Caffeic acid	C_9_H_7_O_4_	179.0341	−4.65	179, **135**	St
9	11.96	3,4-Dicaffeoylquinic acid	C_25_H_23_O_12_	515.1202	1.32	353, 191, 179, **173**, 135	St
10	12.15	1,5-Dicaffeoylquinic acid	C_25_H_23_O_12_	515.1203	1.55	353, **191**	St
11	12.19	Kaempferol 3-*O*-glucoside	C_21_H_19_O_11_	447.0938	1.18	447, 284, 255, **227**	St
12	12.53	3,5-Dicaffeoylquinic acid	C_25_H_23_O_12_	515.1202	1.32	353, **191**, 179, 135	St
13	12.61	Methoxyoxaloyl dicaffeoylquinic acid/Malonyl dicaffeoylquinic acid **	C_28_H_25_O_15_	601.1210	1.9	395, 353,335, **233**, 191, 179, 173, 162	[18,19,20,21]
14	12.86	Isorhamnetin hexoside	C_22_H_21_O_12_	477.1044	1.22	477, 315, 299, **271**, 243	[25]
15	13.98	4,5-Dicaffeoylquinic acid	C_25_H_23_O_12_	515.1201	1.08	353, **191**, 179, 135	St
16	14.77	Methoxyoxaloyl dicaffeoylquinic acid/Malonyl dicaffeoylquinic acid **	C_28_H_25_O_15_	601.1213	2.4	395, 353, **233**, 191, 179, 173, 162	[18,19,20,21]
17	15.26	1,3,5-Tricaffeoylquinic acid	C_34_H_29_O_15_	677.1526	2.15	515, 353, **191**, 179, 161, 135	[18]
18	19.37	1,4,5-Tricaffeoylquinic acid	C_34_H_29_O_15_	677.1527	2.24	515, 353, 191, 179, **173**, 161, 135	[18]
19	19.4	3,4,5-Tricaffeoylquinic acid	C_34_H_29_O_15_	677.1529	2.51	515, 353, 191, **179**, 173, 161, 135	[18]
20	19.55	Methoxyoxaloyl tricaffeoylquinic acid/Malonyl tricaffeoylquinic acid **	C_37_H_31_O_18_	763.1532	2.11	557, 539, 515, **395**, 233, 191, 179, 173, 161, 135	[18,19,20,21]
21	21.26	Hispidulin	C_16_H_11_O_6_	299.0565	1.46	299, **284**	[25]
22	21.31	Methoxyoxaloyl tricaffeoylquinic acid/Malonyl tricaffeoylquinic acid **	C_37_H_31_O_18_	763.1533	2.27	515, **395**, 233, 191, 179, 173, 161, 135	[18,19,20,21]
23	22.37	Dihydroxy-dimethoxyflavone	C_17_H_13_O_6_	313.0721	1.03	313, 298, **283**, 255	[25]

* Bold fragments labeled the base peak ions; ** MS data did not allow for the unambiguous identification; St—identification by direct comparison with the standard.

**Table 3:** Content of caffeoylquinic acids [mg/g DW] in *Arnica montana* shoots elicited with different concentrations of YE.

YE, mg/L	5-CQA	3,4-DCQA	3,5-DCQA	1,5-DCQA	4,5-DCQA	UTCQA	Total
0	0.23 ± 0.01 ^d^	0.08 ± 0.01 ^c^	0.35 ± 0.02 ^c^	1.34 ± 0.02 ^d^	0.09 ± 0.01 ^c^	0.94 ± 0.02 ^c^	3.02 ± 0.10 ^c^
50	0.28 ± 0.01 ^c^	0.08 ± 0.01 ^b,c^	0.28 ± 0.01 ^d^	1.44 ± 0.02 ^c^	0.09 ± 0.01 ^c^	0.96 ± 0.02 ^c^	3.13 ± 0.07 ^c^
100	0.68 ± 0.01 ^a^	0.17 ± 0.01 ^a^	0.61 ± 0.01 ^a^	3.02 ± 0.03 ^a^	0.21 ± 0.01 ^a^	1.77 ± 0.02 ^a^	6.45 ± 0.07 ^a^
200	0.37 ± 0.01 ^b^	0.11 ± 0.01 ^b^	0.42 ± 0.01 ^b^	1.79 ± 0.01 ^b^	0.15 ± 0.01 ^b^	1.32 ± 0.02 ^b^	4.16 ± 0.07 ^b^

Values are means ± SE, n = 12; different letters indicate significant differences assessed by the Fisher LSD test (*p* ≤ 0.05) after performing ANOVA one-way analysis. We used the letter “a” for the highest data value and descended to the next for lower data values.

**Table 4:** Effect of salicylic acid on the growth of *Arnica montana* L. in vitro plantlets.

Nutrient Medium	Mean Number of Shoots Explant^−1^	Mean Height cm Shoots^−1^	Fresh Weight g Shoots^−1^
Control	3.00 ± 0.20 ^a^	1.46 ± 0.09 ^b^	0.32 ± 0.02 ^b^
50 µM SA	2.95 ± 0.23 ^a^	1.56 ± 0.11 ^ab^	0.36 ± 0.03 ^ab^
100 µM SA	2.60 ± 0.25 ^a^	1.96 ± 0.15 ^a^	0.47 ± 0.04 ^a^
200 µM SA	1.70 ± 0.16 ^b^	1.90 ± 0.13 ^ab^	0.38 ± 0.03 ^ab^
LSD	0.61	0.35	0.09

The data are presented as means of 20 shoots per treatment variant ± standard error (SE). Different letters indicate significant differences assessed by the Fisher LSD test (*p* ≤ 0.05) after performing one-way ANOVA. We used the letter “a” for the highest data value and descended to the next for lower data values.

**Table 5:** Content of caffeoylquinic acids [mg/g DW] in *Arnica montana* shoots elicited with different concentrations of SA.

SA, µM	5-CQA	3,4-DCQA	3,5-DCQA	1,5-DCQA	4,5-DCQA	UTCQA	Total
0	0.39 ± 0.01 ^a^	0.07 ± 0 ^a^	0.38 ± 0.01 ^a^	2.07 ± 0.02 ^a^	0.14 ± 0.01 ^a^	1.79 ± 0.03 ^a^	4.85 ± 0.08 ^a^
50	0.28 ± 0.01 ^b^	0.04 ± 0.01 ^b^	0.28 ± 0.01 ^b^	1.30 ± 0.01 ^b^	0.06 ± 0 ^b^	0.66 ± 0.02 ^b^	2.62 ± 0.06 ^b^
100	0.22 ± 0.01 ^c^	0.03 ± 0 ^b,c^	0.23 ± 0.01 ^c^	1.00 ± 0.01 ^c^	0.04 ± 0.01 ^b,c^	0.42 ± 0.01 ^c^	1.94 ± 0.06 ^c^
200	0.19 ± 0 ^c^	0.02 ± 0 ^c^	0.14 ± 0.01 ^d^	0.66 ± 0.02 ^d^	0.03 ± 0 ^c^	0.20 ± 0 ^d^	1.25 ± 0.04 ^d^

Values are means ± SE, n = 12; different letters indicate significant differences assessed by the Fisher LSD test (*p* ≤ 0.05) after performing ANOVA one-way analysis. We used the letter “a” for the highest data value and descending to the next for lower data values.

## Data Availability

Data are contained within the article and Supplementary Materials.

## Supplementary Materials

The following supporting information can be downloaded at: https://www.mdpi.com/article/10.3390/plants14060967/s1, Figure S1: The structures of the main compounds identified in *A. montana* shoots.
