# Supplementary material for: Antioxidant Capacity and Accumulation of Caffeoylquinic Acids in Arnica montana L. In Vitro Shoots After Elicitation with Yeast Extract or Salicylic Acid
Source: Plants (Basel). 2025 Mar 19;14(6):967. doi: 10.3390/plants14060967 (PMC11945374; doi:10.3390/plants14060967)
Supplement: Supplementary file 1 [file plants-14-00967-s001.zip › plants-3502259-supplementary.pdf]

Antioxidant Capacity and Accumulation of Caffeoylquinic Acids in *Arnica montana* L. *In Vitro* Shoots After Elicitation with Yeast Extract or Salicylic Acid

SUPPLEMENTARY MATERIAL

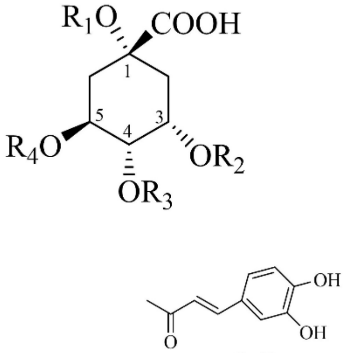

Caff

	R_1	R_2	R_3	R_4
**3-CQA**	H	Caff	H	H
**5-CQA**	H	H	H	Caff
**3,4-DCQA**	H	Caff	Caff	H
**1,5-DCQA**	Caff	H	H	Caff
**3,5-DCQA**	H	Caff	H	Caff
**4,5-DCQA**	H	H	Caff	Caff
**1,3,5-TCQA**	Caff	Caff	H	Caff
**1,4,5-TCQA**	Caff	H	Caff	Caff
**3,4,5-TCQA**	H	Caff	Caff	Caff

Figure S1. The structures of the main compounds identified in *A. montana* shoots
